# 肺转移瘤的外科治疗进展

**DOI:** 10.3779/j.issn.1009-3419.2019.09.04

**Published:** 2019-09-20

**Authors:** 波 刘, 晖 夏

**Affiliations:** 100037 北京，解放军总医院第四医学中心胸心外科 Department of Cardiothoracic Surgery, the Fourth Medical Center of PLA General Hospital, Beijing 100037, China

**Keywords:** 肺肿瘤, 肺转移瘤, 外科治疗, Lung neoplasms, Pulmonary metastases, Surgical treatment

## Abstract

肺是除肝脏以外肿瘤最常见的转移部位，肺转移瘤切除术也是目前胸外科常规手术之一。然而肺转移瘤切除术的临床效果尚存争议，但就目前的临床经验，无瘤间隔时间长、原发肿瘤恶性程度低、转移瘤可被完全切除的肺转移瘤患者行肺转移瘤切术获益最大。本文就肺转移瘤的外科治疗进展进行综述回顾。

## Abstract (en)

Lung is the most common metastatic site for tumors other than the liver. Pulmonary metastasectomy is also one of the common operations in thoracic surgery. However, the effect of pulmonary metastasectomy is controversial. As far as the current experience is concerned, patients with pulmonary metastases with long disease free interval, low-grade malignant tumor and complete excision have the greatest benefit from metastasectomy. This review is about the progress of surgical treatment of pulmonary metastases.

虽然近年来对癌症的系统性治疗有长足进步，但各类型癌症转移瘤的发病率仍持续上升^[1]^。肺是除肝脏外肿瘤最常见转移部位^[2]^，既往认为肿瘤远处转移是手术的禁忌证，但20世纪30年代开始逐渐有小样本的回顾研究报道了肺转移瘤切除术后患者获得较长的生存期，20世纪70年代开始肺转移瘤切除术的意义逐渐得到重新认识，如对孤立病灶的病理鉴定、对化疗无反应病灶的切除等，并逐渐扩大了手术适应证^[3]^。受限于随访时间、样本量、生物学和组织学的异质性等因素，目前关于肺转移瘤的临床研究多为回顾性研究。1997年国际肺转移瘤注册中心报道了一项纳入5, 206例患者的研究，根据原发灶类型分为上皮来源肿瘤、肉瘤、生殖细胞肿瘤、黑色素瘤，指出患者术后生存时间与原发灶术后转移瘤出现的时间间隔、转移灶数量以及转移瘤的可切除性相关^[4]^。结直肠癌肺转移是目前唯一正在进行中的关于肺转移瘤治疗的随机对照试验，旨在比较主动监测与肺转移瘤切除并主动监测的临床效果，预计2020年公布结果^[5]^; 其余类型的肺转移瘤，如黑色素瘤、生殖细胞肿瘤、肉瘤、妇科肿瘤、泌尿系统肿瘤等肺转移瘤，尚无前瞻性研究。

## 1 肺转移瘤切除术

经过长期临床总结，目前对肺转移瘤切除术的适应证及影响预后的因素达成一些共识。手术适应证：（1）原发肿瘤可被控制或已被控制; （2）没有胸外转移存在; （3）所有肿瘤必须可被切除，并有足够的肺功能储备; （4）没有其他更低发病率的替代方案^[6]^。一般有利与预后的特征有：（1）一个或几个转移; （2）长期无病间期; （3）结直肠癌中的正常CEA水平^[2]^。不良预后特征有：（1）活动性原发癌; （2）胸外转移; （3）无法获得手术切除; （4）纵隔淋巴结转移^[2]^。

据统计超过75%的肺转移瘤患者合并有胸外转移，只有约有15%-25%的患者转移灶是局限于肺内且适宜行治愈性切除^[7]^，因此术前检查应包括胸部计算机断层扫描（computed tomography, CT）、腹部CT、头部CT或磁共振成像（magnetic resonance imaging, MRI），必要时行全身正电子发射型计算机断层显像（positron emission computed tomography, PET）/CT检查，以明确肿瘤转移情况。

就手术而言，基本原则是尽可能完整切除转移灶、尽量多的保留肺组织，以提高患者生存率以及改善生存质量。大多数的转移灶位于外周，易于行楔形切除术，部分深部的转移灶无法行楔形切除术的可行解剖性切除，如段切除、肺叶切除等，但应慎重行全肺切除，有报道因肺转移瘤行全肺切除后生存期均未超过2年^[8]^。就术式方面，常规开放手术的优势是更容易发现和切除更多肺内转移灶，尤其是位于肺组织深处的病灶; 电视胸腔镜手术则具有手术切口小、术后疼痛轻、引流及住院时间短、对肺组织表面观察清晰等优势，缺点是不方便直接用手对肺组织进行探查，尤其是部分深处小病灶不易在术中定位发现，但电视胸腔镜依旧是十分安全的术式^[9]^，并且近年研究^[10, 11]^认为电视胸腔镜下肺转移瘤切除术后生存改善并不亚于传统开放手术。

胸内淋巴结侵犯是影响预后的不良因素之一，目前有研究认为结直肠癌和肾细胞癌肺转移的纵隔淋巴结清扫可能有助于提高生存率^[12, 13]^，但对于其余类型肿瘤肺转移的纵隔淋巴结清扫是否可以提高患者的生存率目前尚无充分证据^[14, 15]^。

## 2 结直肠癌肺转移

有研究指出大约有1/4的结直肠癌患者在确诊时存在转移病灶，转移灶最常见于肝脏和肺^[16, 17]^，结直肠癌肺转移瘤切除术后5年生存率约49.4%，双侧肺转移瘤、多个肺转移瘤、Ⅳ期结直肠癌为不良预后因素，而术前血浆CEA水平、CA19-9水平以及原发肿瘤位置与预后无统计学意义，Ⅰ期-Ⅲ期结直肠癌伴单侧肺转移行肺转移瘤切除可最大获益^[16]^。

尽管很多回顾性研究报道了结直肠癌肺转移瘤切除术的临床效果，但目前仍无前瞻性临床研究证实。GLIDA试验指出结直肠癌术后强化监测与最小频率监测对肿瘤复发的预后影响无统计学差异^[18]^，该研究提示结直肠癌术后肺转移瘤的早期诊断并不能改善预后。PulMiCC试验是目前唯一正在进行中的结直肠癌肺转移瘤的前瞻性临床研究，目的在于比较主动监测与肺转移瘤切除并主动监测的临床效果，预计2020年公布研究结果^[5]^。

胸内淋巴结侵犯是结直肠癌肺转移预测不良预后的重要因素之一，有研究指出有必要行淋巴结取样或切除以利于患者预后预测^[19]^，而且结直肠癌肺转移的纵隔淋巴结清扫可能有助于提高术后生存率^[20]^。

## 3 软组织肉瘤和骨肉瘤肺转移

肉瘤包含多种混杂的组织学亚型，具有肺部转移倾向。在软组织肉瘤患者中约有20%存在孤立性肺转移灶，而在骨肉瘤患者中发生肺转移的比例约为40%，甚至3/4的患者在发现骨肉瘤时就存在转移^[21]^。

对于患有软组织肉瘤和骨肉瘤肺转移患者，转移瘤切除术是一种较成熟的治疗方式，有回顾性研究^[22]^分析了372例行肺转移瘤切除的患者，楔形切除约占85%、解剖性肺段切除约占5%、肺叶切除约占10%，行双侧手术的约占30%;术后并发症出现率约2.75%，30 d死亡率为0%;就转移灶个数来说，3个转移灶以下的约占87%，4个-9个转移灶的约占9%，超过10个转移灶的约占4%;肉瘤患者很少有胸内淋巴结转移，且无论转移与否都不是影响预后的因素。

另有研究^[23]^指出肉瘤患者的肺转移瘤切除术的不良预后特征有：肺转移瘤不完全切除、两次手术间隔 < 1年、多于3个转移瘤、肺转移瘤的总最大直径 > 45 mm。

同期有长期回顾性研究^[24]^报道肺转移瘤切除术后1年、3年、5年、10年生存率分别为82.9%、52.2%、28.3%和13.3%，同时指出 < 50岁、病理低恶性程度、无瘤间隔 > 13.5个月是良好预后的重要预测因素。

根据目前大多数回顾性临床研究，对于软组织肉瘤和骨肉瘤肺转移患者，即便对于双侧转移和多发转移患者，肺转移瘤切除术是一项安全可行并可能改善患者预后的有效治疗策略^[25]^。

## 4 头颈部癌肺转移

肺是头颈部癌最常见的远处转移部位，组织学类型包括鳞状细胞癌、腺样囊性癌、疣状癌以及其他未细分类型，头颈部癌肺转移瘤中鳞癌约占75%，且鳞癌相较腺样囊性癌有更低的5年生存率（29.1% *vs* 66.8%）^[26, 27]^。

头颈部癌患者的肺转移瘤常对化疗不敏感，因此转移瘤切除术可能是一项有效的治疗手段^[28]^。有研究^[28, 29]^报道头颈部癌肺转移瘤术后5年总体生存率约为52%-57%，中位生存期约66个月-77个月，不良预后因素包括：鳞癌、无瘤间隔时间 < 18个月、发现肺转移灶前头颈部癌复发、转移瘤直径 > 25 mm、年龄大于60岁，研究提示对于部分头颈部癌肺转移患者（不良预后因素不超过3个）转移瘤切除术有助于提高生存率。

## 5 泌尿系统恶性肿瘤肺转移

泌尿系统恶性肿瘤包括尿路上皮细胞癌和肾细胞癌，尿路上皮细胞癌包括上尿路上皮细胞癌、膀胱癌、尿道癌，而肺是泌尿系统肿瘤最常见的转移部位^[30]^。

尽管化疗对于尿路上皮细胞癌转移瘤有较高初始响应率，但中位总生存期只有14个月-15个月; 而研究报道尿路上皮细胞癌转移瘤切除术后中位总生存期约为30个月，3年生存率约为41%，并指出转移瘤切除术可能有助于尿路上皮细胞癌转移瘤患者获得更良好的预后，尤其对于是单个转移灶、肺转移以及肿瘤复发患者^[31]^，然而该研究同样指出转移瘤切除术只是延长了肿瘤进展时间，对总体生存率并无显著影响。但近期有研究^[32]^指出膀胱癌合并单个转移灶患者行转移瘤切除术有助于延长总生存期，尤其是对于年龄 < 65岁患者和肌肉浸润性膀胱癌患者。

肾细胞癌肺转移瘤切除术的临床效果虽然没有前瞻性研究，但根据现有临床数据其治疗效果基本得到了认可。有研究报道术后5年总体生存率约为33.4%，中位生存期为39.2个月，肺转移灶是否完全切除是影响预后的关键因素，完全切除者5年生存率为39.9%、中位生存期为46.6个月，不完全切除者则分别为0%和13.3个月; 此外，异时转移的5年生存率优于同时转移（43.7% *vs* 0%），单转移灶的5年生存率优于多个转移灶（49% *vs* 23%）; 研究提示对于肾细胞癌肺转移患者，转移瘤切除术可能是目前的最佳治疗选择^[33, 34]^。

## 6 妇科恶性肿瘤肺转移

妇科恶性肿瘤出现肺转移的发生率约为2.3%-4.6%，肉瘤和绒毛膜癌较宫颈癌、子宫内膜癌、卵巢癌等上皮来源肿瘤更易发生肺转移^[35]^。

有研究^[36]^报道妇科恶性肿瘤肺转移瘤切除术后5年和10年总体生存率分别为40.9%和31.4%，而经过辅助治疗的患者5年生存率约为52.4%，无瘤时间间隔 < 24个月，肺转移瘤切除术后复发是不良预后预测因素。

对于妇科恶性肿瘤肺转移患者，可能受限于样本量小的原因，目前的研究^[35]^显示肺转移瘤切除术和化疗的5年生存率尚无显著统计学差异，但前者的5年总体生存率有高于后者的趋势（89.1% *vs* 49.5%, *P*=0.072），提示手术切除孤立性肺转移瘤（< 3个）可能对无复发间隔时间长的患者、化疗耐药患者以及肿瘤再复发患者的预后提供潜在的益处。

## 7 黑色素瘤肺转移

肺通常是黑色素瘤最先发生远处转移的部位^[37]^，而对于部分肺转移患者，转移瘤切除术目前被认为是一种重要的治疗措施^[38, 39]^。研究报道黑色素瘤肺转移切除术后5年生存率约为18%-39.4%、中位生存期为17个月-24个月^[40]^，良好预后因素包括：转移病变大小不超过23 mm、转移灶完整切除、转移瘤个数（不超过3个）^[37, 38]^。但有研究报道现阶段各种单克隆抗体的应用也可将黑色素瘤肺转移患者中位生存期延长至17个月以上^[40]^，因此黑色素瘤肺转移瘤切除术的意义再次受到质疑，有待进一步研究。

## 8 乳腺癌肺转移

肺是复发性乳腺癌常见的转移部位，但介于目前内分泌治疗、化疗及靶向治疗等非手术方式常可获得理想疗效，因此对乳腺癌肺转移瘤切除术的价值尚存在较大争议^[41, 42]^。

有研究报道单纯肺转移的患者约占乳腺癌转移患者中的10%-20%，单纯行化疗的患者5年生存率只有2.4%^[43]^; 而行肺转移瘤切除术的患者5年总体生存率约为27%-54%，中位生存期约为32个月-97个月^[44]^，不良预后预测因素包括：无肿瘤间隔时间 < 3年、肺转移瘤不完全切除、肺转移瘤个数超过1个、转移瘤HER2受体阴性^[45, 46]^。然而，上述均是不同时间独立的回顾性研究结果，因此无法辨别研究结果差异究竟是转移瘤手术的获益还是患者选择造成的偏倚，目前仍缺乏将转移瘤切除术与非手术方式治疗直接进行比较的临床研究。

## 9 总结

肺转移瘤的外科治疗一直是存在争议的领域，1990年国际肺转移瘤注册中心的建立极大的推动了肺转移瘤的临床研究。受限于肺转移瘤的病例随访时间短、纳入病例偏少以及肿瘤组织学的异质性，目前尚缺乏关于肺转移瘤的大样本临床对照试验结果，因此肺转移瘤的外科治疗仍存在诸多争议。但就目前的临床经验，无瘤间隔时间长、原发肿瘤恶性程度低、转移瘤可被完全切除的肺转移瘤患者行肺转移瘤切术可能获益最大。
